# Validation of Cardiorespiratory Fitness Measurements in Adolescents

**DOI:** 10.3390/jfmk4030044

**Published:** 2019-07-13

**Authors:** Pedro Migliano, Laura S. Kabiri, Megan Cross, Allison Butcher, Amy Frugé, Wayne Brewer, Alexis Ortiz

**Affiliations:** 1 School of Physical Therapy, Texas Woman’s University, 6700 Fannin, Houston, TX 77030, USA; 2 Department of Kinesiology, Rice University, 6100 Main Street, Houston, TX 77005, USA; 3 Department of Physical Therapy, UT Health San Antonio, 7703 Floyd Curl Dr. San Antonio, TX 78229, USA

**Keywords:** VO_2max_, PACER, non-exercise test

## Abstract

Cardiorespiratory fitness (CRF) is an important indicator of adolescent cardiovascular well-being and future cardiometabolic health but not always feasible to measure. The purpose of this study was to estimate the concurrent validity of the non-exercise test (NET) for adolescents against the Progressive Aerobic Capacity Endurance Run (PACER^®^) and direct measures of VO_2max_ as well as to examine the concurrent validity of the PACER^®^ with a portable metabolic system (K4b^2™^). Forty-six adolescents (12–17 years) completed the NET prior to performing the PACER^®^ while wearing the K4b^2™^. The obtained VO_2max_ values were compared using linear regression, intra-class correlation (ICC), and Bland–Altman plots, and α was set at 0.05. The VO_2max_ acquired directly from the K4b^2™^ was significantly correlated to the VO_2max_ indirectly estimated from the NET (*r* = 0.73, *p* < 0.001, *r*^2^ = 0.53, ICC = 0.67). PACER^®^ results were significantly related to the VO_2max_ estimates from the NET (*r* = 0.81, *p* < 0.001, *r*^2^ = 0.65, ICC = 0.72). Direct measures from the K4b^2™^ were significantly correlated to the VO_2max_ estimates from the PACER^®^ (*r* = 0.87, *p* < 0.001, *r*^2^ = 0.75, ICC = 0.93). The NET is a valid measure of CRF in adolescents and can be used when an exercise test is not feasible.

## 1. Introduction

Low cardiorespiratory fitness (CRF) levels in adolescents have been linked to insulin resistance as well as cardiovascular and cardiometabolic risk factors [1,2,3,4,5]. Higher levels of CRF in adolescents have also been linked to improved academic outcomes and an improved ability to regulate attention and behavior [6,7]. There is a strong positive relationship between CRF and cognitive performance, with multiple studies showing that adolescents with higher levels of CRF outperformed their peers with lower levels of CRF in cognitive tests involving working memory, cognitive flexibility, and inhibition, which is the ability to ignore extraneous environmental information [8,9,10]. The improved cognitive ability is thought to be due to the increased volume in different regions of the brain such as the hippocampus and basal ganglia found in adolescents with higher levels of CRF [8,11]. Moreover, higher CRF has been shown to be an excellent predictor of physical activity level during adulthood [12,13]. Since CRF is an important health predictor in adolescents, it should be regularly assessed in both healthcare and school settings.

Maximal oxygen uptake (VO_2max_), expressed as mL/kg/min of oxygen, is the gold standard measure of CRF and can be measured both directly and indirectly [14,15]. A direct measure of VO_2max_ is obtained by ventilatory gas analysis during a graded exercise test at maximum exertion and is the most precise measure of VO_2max_ [14]. An indirect measure estimates VO_2max_ from a maximal or submaximal exercise test and is usually estimated from total time, total work, or heart rate [15]. Especially in school settings, VO_2max_ is often indirectly measured and estimated using a field test. While there are a few different options when it comes to field tests to indirectly measure VO_2max_, the 20-m multistage shuttle run test (20MSR) is the most frequently used test, as it has shown moderate to high criterion validity compared to direct measures of VO_2max_ and is a more practical test compared to longer duration endurance tests, such as the 6-min endurance run [16,17]. The Progressive Aerobic Cardiovascular Endurance Run (PACER^®^) is part of the FitnessGram^®^ tests administered to adolescents in schools [18]. The PACER^®^ is a 20MSR meant to mimic a maximal exertion exercise test with workloads increasing every stage until the participant reaches volitional exhaustion. The standardized procedure of the PACER^®^ has participants run between two markers placed 20 m apart while keeping pace to a prerecorded audio cadence that increases every minute. The test is terminated if a participant fails to reach a marker for the second time or if the participant can no longer continue. This field test has been shown to be both reliable (intra-class correlation (ICC): 0.77–0.93) and valid (*r*: 0.62–0.83) [16,19,20], when compared to directly measured VO_2max_ during a treadmill maximal exercise test. 

It is not always possible to directly assess VO_2max_ in all settings, such as schools, gyms, or even in a clinical setting. Ventilatory gas analysis is time-consuming and requires expensive equipment that prevents the mass screening which would be required in a school setting or in the daily caseload and time restraints of a healthcare professional. The non-exercise test (NET) is a paper-based questionnaire used to estimate VO_2max_ from different variables including sex, age, body mass index (BMI), resting heart rate, and self-reported habitual physical activity levels [21,22]. Use of this questionnaire allows for both quick and widespread CRF screening without any health risk to the participants. The NET has been shown to be valid within the adult population [21], but to the best of our knowledge, has not been tested within the adolescent population. Therefore, the purpose of this study was to estimate the concurrent validity of the NET for adolescents against the PACER^®^ test and objective measures of VO_2max_ as well as to examine the concurrent validity of the VO_2max_ estimated by the PACER^®^ with the VO_2max_ obtained from the Cosmed K4b^2™^ portable metabolic system (Cosmed K4b^2^, Cosmed, Rome, Italy) during the PACER^®^ test.

## 2. Materials and Methods

### 2.1. Subjects

Adolescents aged 12–17 years were recruited by email, education support groups, co-operatives, and word of mouth. Parents were asked if their child had any physical or mental limitations that would prevent them from safely and accurately completing the test and if any were noted, the child was excluded. Institutional review board approval (Protocol #19736 on 19 January 2017) from Texas Woman’s University in Houston, TX, parental informed consent, and minor assent were secured prior to any subject enrollment or data collection.

### 2.2. Procedures

Height and weight were assessed barefoot with light clothing using a medical-grade stadiometer calibrated prior to its use. Pubertal level was assessed using the pubertal developmental scale.

Prior to participating in the PACER and being fitted with the K4b^2™^, participants were given the NET questionnaire previously validated in adults by Jurca et al. [21]. As required by the NET, each participant was asked to choose the physical activity level which best described their daily level of activity, as listed in Table 1. Participant BMI was calculated based on their weight and height measurement. Resting heart rate (RHR) was measured with the SantaMedical SM150BL finger pulse oximeter (SantaMedical, Tustin, CA, USA) as the final measurement after sitting for at least five minutes.

The K4b^2™^ portable metabolic system was used to directly measure VO_2max_ during the PACER^®^. The K4b^2™^ has demonstrated high test–retest reliability (ICC = 0.70–0.90) [23] and has shown to be valid in VO_2_ measurement when compared to a traditional stationary gas exchange system (*r*: 0.93–0.97) [24]. The K4b^2™^ was calibrated according to manufacturer’s recommendations using known gases and room air sampling. After calibration, participants were fitted with a mask and connected to the K4b^2™^ prior to initiating the PACER^®^.

After donning the K4b^2™^, adolescents were allowed to wear and familiarize themselves with the device for 5 min. Participants completed the 20-m PACER^®^ individually as per the standardized instructions. Two pieces of tape were placed 20 m apart, denoting the crossing lines for the PACER^®^, and the FitnessGram^®^ cadence soundtrack was played at a volume loud enough for the participant to hear. Participants were instructed to begin running when they heard the first beep on the soundtrack. For the lap to be valid, each participant was required to clear at least one foot over the line prior to the next beep. The test was terminated following the second miss or if the participant desired to stop. All raters were trained and followed the standardized test procedures. Once the PACER^®^ had been terminated, total laps were recorded, the K4b^2™^ data was saved, and the mask was doffed. 

VO_2max_ with the K4b^2™^ system was determined in the same manner used by Silva et al. [25]. Respiratory variables were recorded breath-by-breath and averaged over a 10-s period. VO_2max_ was determined when a plateau in the VO_2_ curve was detected and if the plateau was absent the VO_2peak_ was taken instead. VO_2max_ with the PACER^®^ was calculated using the quadratic model established by Mahar, Guerieri, Hanna, and Kemble [26]: VO_2max_ = 41.76799 + (0.49261 × laps) − (0.00290 x laps^2^) − (0.61613 × BMI) + (0.34787 × sex × age), where sex is 0 for males and 1 for females.

### 2.3. Statistical Analyses

The Shapiro–Wilk test, Levene’s test, and box plots were utilized to screen all data for normality assumptions, homoscedasticity, and outliers, respectively. Linear regressions with Pearson correlations were performed on the VO_2max_ estimates acquired from the PACER^®^ and the NET, and the VO_2max_ acquired from the K4b^2™^ system. Intra-class correlations (ICCs), standard error of estimate (SEE), paired sample *t*-tests, and Bland–Altman plots were performed to assess agreement and differences between the measures of VO_2max._ All statistical analyses were conducted with IBM SPSS software for Windows (v. 25.0; IBM Corp., Armonk, NY, USA). 

## 3. Results

Forty-six adolescents (male: 24; female: 22) were recruited. All data met all assumptions for normality, homoscedasticity, and outlier assumptions. Sex specific demographic and anthropometric characteristics are listed in Table 2.

**Figure 1:**
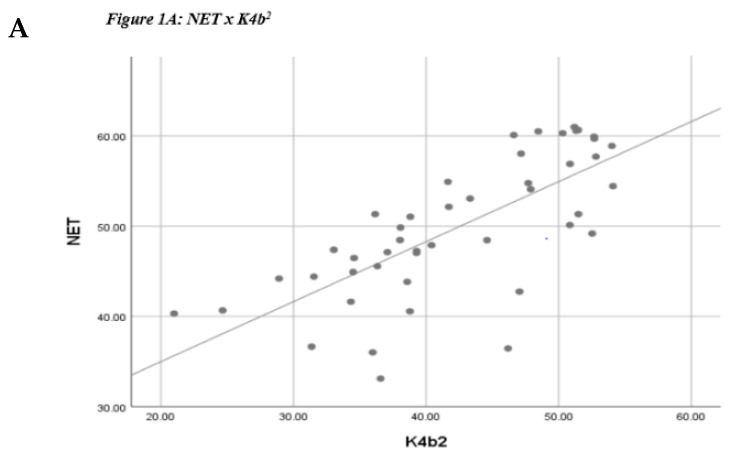

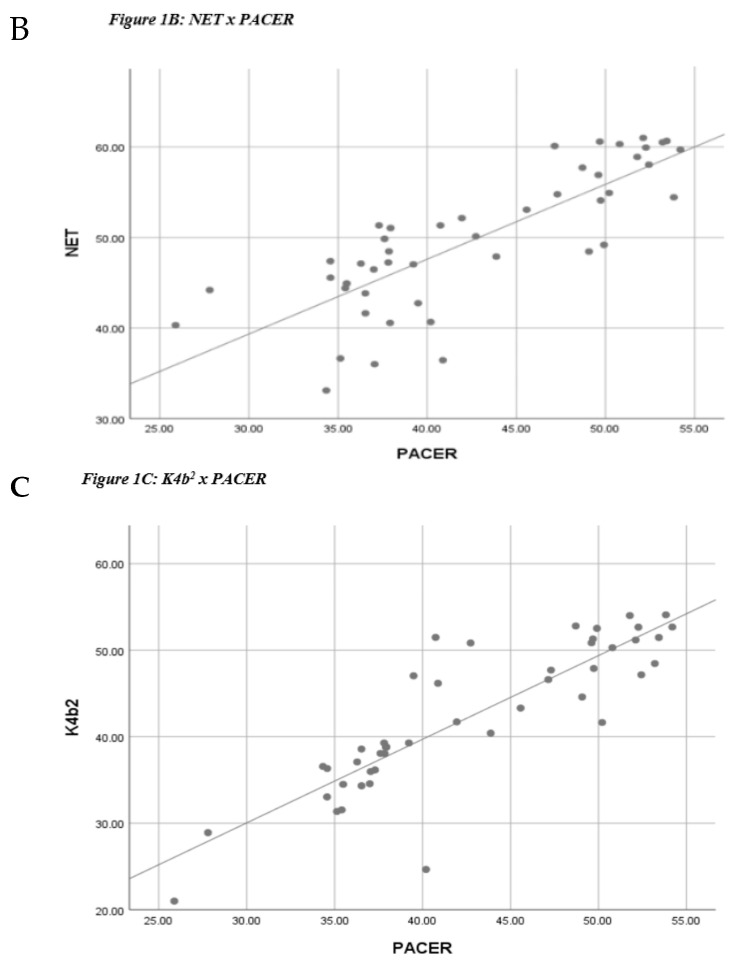
Simple scatterplots. (**A**) NET & K4b^2^: *r* = 0.73; (**B**) NET & PACER^®^: *r* = 0.81; (**C**) K4b^2^ & PACER^®^: *r* = 0.87; Bland–Altman plots (Figure 2) and paired sample *t*-tests demonstrated acceptable limits of agreement and no significant difference for K4b^2™^ and PACER^®^ measures of VO_2max_ (Figure 2A) (mean difference = −0.37, *t*(45) = −0.61, *p* = 0.55) but showed that the NET VO_2max_ estimates tend to be overestimated, seen by the upward shift of the mean difference line away from zero, and significantly different in the NET − PACER^®^ (Figure 2B) (mean difference = 7.14, *t*(45) = 10.26, *p* < 0.001) and the NET − K4b^2™^ (Figure 2C) (mean difference = 7.52, *t*(45) = 8.54, *p* < 0.001) plots.

Pearson correlation coefficients as well as ICC and SEE values are listed in Table 3. Scatterplots depicting the correlations are listed in Figure 1. All assessment methods demonstrated moderate to strong statistically significant correlations and levels of agreement as defined by Portney and Watkins [27] for correlations and Koo and Lee [28] for ICC. 

## 4. Discussion

The aim of this study was to estimate the concurrent validity of the NET for adolescents against the PACER^®^ and objective measures of VO_2max_ as well as to examine the concurrent validity of the VO_2max_ estimation by the PACER^®^ with the VO_2max_ from the K4b^2™^ portable metabolic system during the PACER^®^ test. Past studies have shown that the NET is a valid measure of CRF in adult populations [21,22], and our results show that the NET is a valid predictor of VO_2max_ in the adolescent population. This is important, as it offers a quick, easy, inexpensive, and risk-free method of assessing CRF in adolescents. To our knowledge, this is the first report to measure CRF in adolescents without requiring any form of exertion from the participant, which allows for widespread use among large groups and offers a feasible tool for CRF measurement in healthcare settings. 

The PACER^®^ test has been validated several times [16,19,20], and our results comparing it to the direct measure of VO_2max_ from the K4b^2™^ (*r* = 0.87) align with past results (*r* = 0.62–0.83) acquired directly from treadmill maximal exercise tests [16,19,20]. Our study offers a different perspective, as most studies compare the PACER^®^ estimate to a traditional treadmill maximal exercise test, but our research compared the estimate to the direct measure of VO_2max_ acquired at the same time the participant was performing the PACER^®^. This further strengthens the validity of the PACER^®^ as an indirect measure of VO_2max_ and shows that the PACER^®^ protocol is a valid substitute for a maximally graded exercise test. 

In practice, the NET could offer schools a quick and inexpensive tool to assess CRF more frequently than the PACER^®^ test allows, providing valuable information to implement changes in physical education. At the clinical level, the NET can be implemented into healthcare settings as a snapshot of current CRF and can be used as a tool to assess risk for cardiovascular and cardiometabolic disease [1,2,3,4,5]. For efficient use in either setting, the NET results can be analyzed similar to the PACER^®^, which groups students based on having sufficient or insufficient CRF into the healthy fitness zone (HFZ), the needs improvement zone (NIZ), or the health risk zone (HRZ) [18]. Based on our results, the group values for the zones could be adjusted with the linear regression equation: PACER^®^ = 10.92 + 0.96(NET). For example, a 10-year-old male with a NET score of 46.82–49.42 would fall into the NIZ, a score of ≤46.81 would put him in the HRZ, and a score ≥49.43 would place him into the HFZ. This would allow for a quick assessment to deduce whether an adolescent patient is at risk and requires intervention.

As seen with the Bland–Altman plots and *t*-tests, the NET VO_2max_ estimates were generally much higher and were significantly different than the values acquired directly through the K4b^2™^ or estimated through the PACER^®^. This could be due to the fact the descriptions of the physical activity levels were intended for adults, so it may not have been directly relatable to adolescents. Also, the added weight of the K4b^2™^ unit, weighing roughly 1 kg, plus the addition of the mask could have resulted in a decreased outcome during the PACER^®^ test. While the NET measures of VO_2max_ were significantly higher, the moderately strong levels of agreement show that the NET is valid and by using the CRF grouping suggested above, as is used by the PACER^®^, the NET overestimation is inconsequential. 

Limitations of this study include a small sample of females and males, thus limiting the ability to fully explore sexes separately, and the running of the PACER^®^ test individually instead of in groups, as is the norm in schools, thus potentially affecting participants’ motivation to maximally exert themselves. Regardless of the limitations of this study, there are several strengths in our design and findings, including validating the NET against two different validated measures of VO_2max_ and testing a representative sample of adolescents. Future studies on using the NET for adolescents could examine a larger sample size, perhaps in public schools, to compare against the PACER^®^ or investigate the NET’s sensitivity to change in an adolescent’s CRF. Future studies need to also assess the reliability of the NET in adolescents, so it can be established as a tool to measure CRF over time.

## 5. Conclusions

This article gives researchers, clinicians, and coaches a simple, quick, and inexpensive tool to indirectly estimate VO_2max_ in the adolescent population as opposed to a time-consuming and exhausting exercise test. This article also strengthens the evidence for use of the PACER^®^ test as an indirect measure of VO_2max_, as it provided ventilatory gas analysis during the test itself, showing that the PACER^®^ is a valid graded maximal exertion exercise test, as direct measures of VO_2max_ acquired during performing the PACER^®^ were in agreement with PACER^®^ estimates based on regression equations established against the more traditional treadmill VO_2max_ tests.

## Figures and Tables

**Figure 2:**
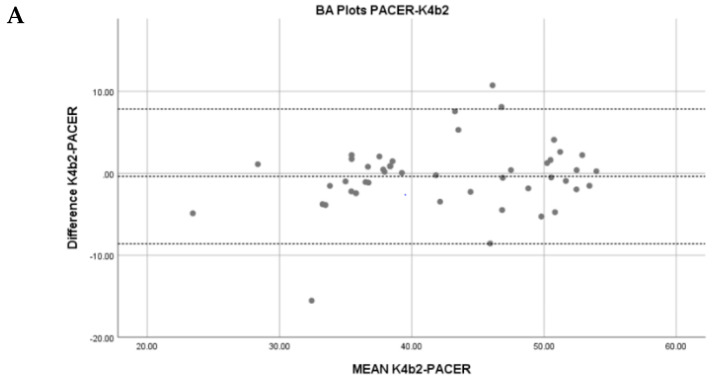

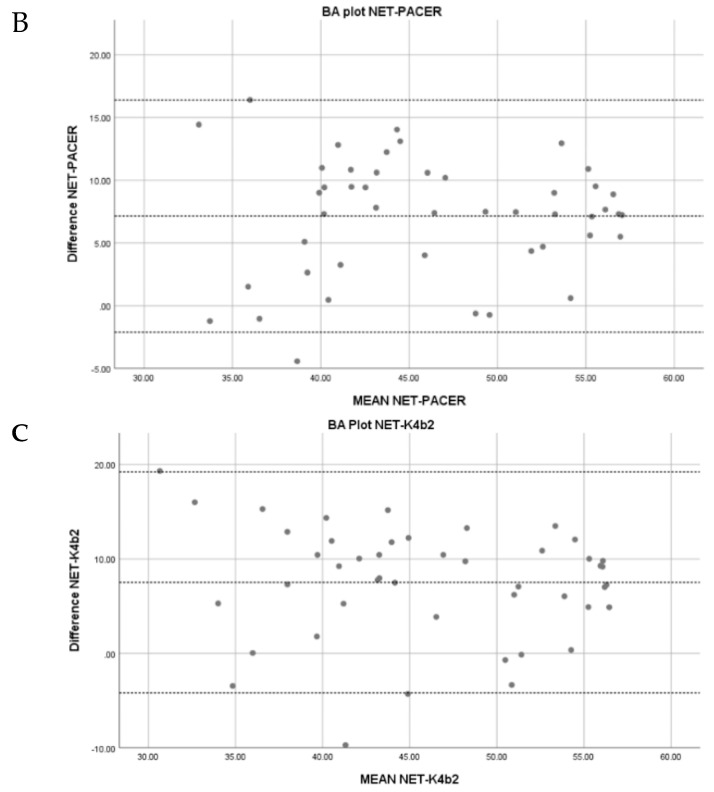
Bland–Altman plots. Middle line represents mean difference while other dotted lines represent the limits of agreement: ±1.96*s*. (**A**) K4b^2^ & PACER^®^: mean difference = −0.37, limits of agreement of −8.61 & 7.85; (**B**) NET & PACER^®^: mean difference = 7.14, limits of agreement of −2.10 & 16.39; (**C**) K4b^2^ & NET: mean difference = 7.52, limits of agreement of −4.19 & 19.23.

**Table 1:** Physical Activity Levels for NET.

Physical Activity Level	Description	Score
1	Inactive or little activity other than usual daily activities.	0.00
2	Regularly (≥5 d/wk) participate in physical activities requiring low levels of exertion that result in slight increases in breathing and heart rate for at least 10 min at a time.	0.32
3	Participate in aerobic exercises such as brisk walking, jogging or running, cycling, swimming, or vigorous sports at a comfortable pace or other activities requiring similar levels of exertion for 20 to 60 min per week.	1.06
4	Participate in aerobic exercises such as brisk walking, jogging or running at a comfortable pace, or other activities requiring similar levels of exertion for 1 to 3 h per week.	1.76
5	Participate in aerobic exercises such as brisk walking, jogging or running at a comfortable pace, or other activities requiring similar levels of exertion for over 3 h per week.	3.03

NET equation: VO_2max_ = 3.5 * ((Sex * 2.77) − (age * 0.1) − (BMI*0.17) − (RHR * 0.03) + (physical activity score * 1.00) + 18.07); Sex: 0 = F, 1 = M.

**Table 2:** Demographic and anthropometric characteristics.

Variables	All Participants (*n* = 46) Mean ± SD [range]
Age (years)	14.56 ± 1.69; [12.00–18.00]
Height (cm)	165.65 ± 10.05; [147.00–188.00]
Weight (kg)	60.44 ± 13.57; [38.20, 93.80]
BMI (kg/m^2^)	21.88 ± 3.91; [16.20–33.40]
BMI Percentiles (%)	61.89 ± 26.35; [9.00, 98.00]
Bodyfat % (DXA)	24.27 ±8.35; [13.20, 45.10]
Pubertal Level (PDS)	2.71 ± 0.81; [0.00, 4.00]
K4b^2^ VO_2max_ (mL/kg/min)	42.29 ± 8.37; [20.99, 54.09]
PACER VO_2max_ Estimate (mL/kg/min)	42.67 ± 7.49; [25.88, 54.20]
NET VO_2max_ Estimate (mL/kg/min)	49.82 ± 7.67; [33.11, 60.99]

**Table 3:** Correlation matrix between oxygen consumption estimates per test for both sexes combined.

Correlations			
	PACER	NET	K4B^2^
PACER			
NET	*r* = 0.81; *p* < 0.001 *r*^2^ = 0.65 ICC = 0.72 SEE = 8.53 mL/kg/min [0.57, 0.99]		
K4B^2^	*r* = 0.87; *p* < 0.001 *r*^2^ = 0.75 ICC = 0.93 SEE = 4.17 mL/kg/min [0.70, 1.0]	*r* = 0.73; *p* < 0.001 *r*^2^ = 0.53 ICC = 0.67 SEE = 9.56 mL/kg/min [0.50, 0.97]	
